# Effect of amoxicillin‐clavulanic acid on clinical scores, intestinal microbiome, and amoxicillin‐resistant *Escherichia coli* in dogs with uncomplicated acute diarrhea

**DOI:** 10.1111/jvim.15775

**Published:** 2020-04-23

**Authors:** Melanie Werner, Jan S. Suchodolski, Reinhard K. Straubinger, Georg Wolf, Jörg M. Steiner, Jonathan A. Lidbury, Felix Neuerer, Katrin Hartmann, Stefan Unterer

**Affiliations:** ^1^ Clinic of Small Animal Internal Medicine, Centre for Clinical Veterinary Medicine Ludwig‐Maximilians‐University Munich Germany; ^2^ Gastrointestinal Laboratory, Department of Small Animal Clinical Sciences, College of Veterinary Medicine and Biomedical Sciences Texas A&M University College Station Texas USA; ^3^ Department of Veterinary Sciences Institute of Infectious Diseases and Zoonoses, Faculty of Veterinary Medicine, Ludwig‐Maximilians‐University Munich Germany; ^4^ Clinic of Small Animal Medicine Ismaning Ismaning Germany

**Keywords:** antibiotic, antimicrobial, canine, diarrhea, resistance, sensitivity

## Abstract

**Background:**

Despite limited evidence of efficacy, antibiotic treatment is still frequently prescribed in dogs with uncomplicated acute diarrhea (AD).

**Objective:**

To assess whether amoxicillin‐clavulanic acid has a clinical benefit, an effect on the fecal microbiome, and the proportion of amoxicillin‐resistant *Escherichia coli* in dogs with AD.

**Animals:**

Sixteen dogs with AD of <3 days duration.

**Methods:**

Prospective, placebo‐controlled, double‐blinded study. Clinical scores were compared between client‐owned dogs randomly assigned to an antibiotic (AG) or a placebo (PG) group. The intestinal microbiome was analyzed using quantitative PCR assays. Amoxicillin‐resistant fecal *E. coli* were assessed semiquantitatively with microbiological methods.

**Results:**

There was no difference in clinical recovery between treated dogs or controls (CADS index day 10: AG group median: 2 (range: 1‐3; CI [1.4; 2.6]); PG group median: 1.6 (range: 1‐3; CI [1.1; 2.4]); *P* > .99). All dogs gained normal clinical scores (CADS index ≤3) after 1 to 6 days (median 2 days) after presentation. There was no significant difference in the fecal dysbiosis index (during treatment: AG mean −2.6 (SD 3.0; CI [−5.1; 0.0]); PG mean −0.8 (SD 4.0; CI [−4.2; 2.5]; *P* > .99) or its bacterial taxa. The proportion of resistant fecal *E. coli* increased (to median: 100%; range: 35%‐100%) during treatment with amoxicillin‐clavulanic acid and was still increased (median: 10%; range 2%‐67%) 3 weeks after treatment, both of which were significantly higher proportions than in the placebo group for both time points (during treatment AG median 100% versus PG median 0.2% (*P* < .001); after treatment AG median 10% versus PG median 0.0% (*P* = .002)).

**Conclusions and Clinical Importance:**

Our study suggests that treatment with amoxicillin‐clavulanic acid confers no clinical benefit to dogs with AD, but predisposes the development of amoxicillin‐resistant *E. coli*, which persist for as long as 3 weeks after treatment. These findings support international guideline recommendations that dogs with diarrhea should not be treated with antimicrobials unless there are signs of sepsis.

**Abbreviations**
AG: antibiotic group
AHDS: acute hemorrhagic diarrhea syndrome
CADS‐Index: Canine Acute Diarrhea Severity Index
PG: placebo group

## 1 INTRODUCTION

Diarrhea is a common reason for dogs being presented for veterinary care with approximately 7% of the dogs presented in small animal practice showing diarrhea.^1^ Uncomplicated acute diarrhea (AD) in dogs is most frequently associated with dietary indiscretion, adverse reactions to food, endoparasites, or transient uncomplicated bacterial/viral infections.^2^, ^3^, ^4^, ^5^, ^6^ In many cases the etiology cannot be identified. This is usually not a problem, because clinical signs typically resolve spontaneously and usually do not recur.^2^, ^4^, ^7^, ^8^, ^9^ International guidelines recommend that in dogs with diarrhea, antimicrobials should only be administered to dogs manifesting systemic signs of illness.^10^, ^11^, ^12^, ^13^, ^14^ Despite these recommendations, it is common that dogs with AD receive an untargeted, short‐term antibiotic course as first‐line medication. Two surveys including 11 060 and 371 dogs performed in Europe showed that between 63% and 71% of dogs with AD were treated with antimicrobials, respectively.^15^, ^16^ Our study group previously evaluated the effect of amoxicillin‐clavulanic acid in dogs with acute hemorrhagic diarrhea syndrome (AHRD) without signs of sepsis, and were unable to show a clinical benefit of antibiotic therapy.^17^, ^18^ A second study in dogs with AHDS revealed that the additional application of metronidazole to amoxicillin‐clavulanic acid did not improve the clinical outcome.^18^

Antibiotic treatment can lead to various negative short‐term effects, such as vomiting, diarrhea, and anorexia.^19^, ^20^ Data from human medicine suggest that dysbiosis induced by antimicrobials is associated with an increased risk for developing asthma, postinfectious irritable bowel syndrome and chronic enteropathies such as Crohn's disease.^21^, ^22^, ^23^, ^24^, ^25^ Antibiotics cause prolonged intestinal dysbiosis and lead to changes in the microbial metabolism pathways in healthy dogs and humans.^26^, ^27^, ^28^, ^29^ Disruption of the intestinal microbiota as a consequence of antibiotic use can lead to life‐threatening *Clostridiodes difficile* infections in humans.^30^ Furthermore, antibiotic treatment leads to advantages in growth for resistant bacteria and provokes the development of new resistance mechanisms.^31^, ^32^, ^33^, ^34^, ^35^

Although antibiotics have been routinely used in dogs with uncomplicated AD over decades, evidence‐based studies documenting any clinical benefit of antibiotic treatment in dogs are sparse^36^ and do not exist for potentiated penicillins, which are frequently used as first‐line antibiotic treatment. Thus, the aims of this study were to evaluate the clinical benefit of amoxicillin‐clavulanic acid, to evaluate the effect of the amoxicillin‐clavulanic acid on the intestinal microbiome, and the proportions of resistant fecal *Escherichia coli* in dogs with uncomplicated AD.

## 2 MATERIALS AND METHODS

### 2.1 Animals

This study was designed as a prospective, double‐blinded, randomized, placebo‐controlled, trial. The study design was approved by the ethical committee of the Centre for Clinical Veterinary Medicine Ludwig‐Maximilians‐University, Munich (reference 68‐19‐05‐2016). An informed client consent form was signed by all owners. The dogs were recruited between July 2016 and January 2018 by the same veterinarian (MW) across 4 small animal clinics in Munich, Germany. The randomization list was formed before start of the study through a third person using a research randomizer available on the following website: https://www.graphpad.com/quickcalcs/randomize1.cfm. Dogs of either sex with acute nonhemorrhagic diarrhea between 5 and 40 kg bodyweight, and of at least 9 months of age were enrolled into this study. Only dogs with a fecal consistency score of at least 2 on the CADS‐Index (Table 1) and a duration of gastrointestinal symptoms of <3 days were enrolled. Exclusion criteria were the following: treatment with an antimicrobial within 30 days or treatment with an anti‐inflammatory drug within 7 days before presentation, blood in feces, any signs of systemic inflammation (eg, rectal temperature > 39.0°C [102.2 °F]), severe illness (eg, lethargic mental status, moderate to severe abdominal pain), or significant dehydration prompting hospitalization. These exclusion criteria were chosen to define the disease as “uncomplicated,” thus only dogs that could be treated as outpatients, were included.

**TABLE 1:** Scoring system of the Canine Acute Diarrhea Severity Index

Activity	0: Normal	1: Mild	2: Moderate	3: Severely decreased
Appetite	0: Normal	1: Mild	2: Moderate	3: Severely decreased
Vomiting	0: normal	1: 1×/d	2:2‐3×/d	3: > 3×/d
Fecal consistency	0: Normal	1: Moist, shaped	2: Pasty	3: Watery diarrhea
Frequency of defecation	0: Normal	1:2‐3×/d	2:4‐5×/d	3: > 5×/d

The histories of all dogs were recorded in a standardized method with specific questions regarding stress‐related factors, diet, dietary changes, feeding of treats, chronic illnesses, other diseases in the last months before presentation, drug administration and timing of any past diarrheic episode.

Diagnostic work‐up in all dogs before inclusion consisted of a blood count, serum chemistry profile (urea nitrogen, creatinine, SDMA, sodium, chloride, potassium, phosphate, total bilirubin, ALT, ALP, gamma‐GT, AST, GLDH, total protein, albumin, globulin, glucose, alpha‐amylase, lipase, cholesterol, fructosamine, creatine kinase, LDH, calcium, magnesium, triglycerides), and a fecal flotation.

### 2.2 Treatment

All dogs were randomly assigned to the antibiotic (AG) or the placebo group (PG) by means of a computer‐generated schedule. Dogs allocated to AG group received capsules filled with amoxicillin‐clavulanic acid per os at 12.5 to 25 mg/kg q12h and dogs allocated to PG group received capsules filled only with the carrier substance lactose (300 mg per capsule) per os q12h. Duration of treatment was 7 days in either group. For blinding purposes, the capsules were indistinguishable between the 2 groups. Every dog received a certain number of capsules according to the predefined weight group. To ensure standardization, all dogs were treated with the same symptomatic treatment: maropitant (Cerenia, Zoetis GmbH, Berlin, Germany) as an antiemetic (1 mg/kg given once subcutaneously) and metamizole (Novaminsulfon, Ratiopharm, Ulm, Germany) as an analgesic (30 mg/kg per os q8h for 2 days). All dogs were fed with the same gastrointestinal diet (Royal Canin Gastrointestinal wet or dry) for 7 days.

### 2.3 Evaluation of treatment efficacy

Particular attention was paid to the clinical improvement over time. At the day of presentation and inclusion into the study, defined as day 0, all dogs were evaluated with the canine acute diarrhea severity (CADS) index (Table 1). Every following day, from day 1 to day 10, the score was reassessed by the owner and documented in a diary. If dogs defecated more than once per day, the fecal consistency score was calculated as the average of all defecations of that day. To evaluate the difference between groups, the CADS‐Index of each day was compared between AG and PG. Moreover, the time to normalization of fecal consistency (score 0 or 1) was documented in every individual dog.

### 2.4 Sample collection

Naturally passed fecal samples from each dog were collected by the clinician on day 0 before starting the treatment, and by the owner on day 6 and day 30. Samples for fecal flotation (performed on days 0 and 6) and bacterial culture (performed on days 0, 6, and 30) were processed within a few hours after collection (ie, <6 hours), whereas aliquots for microbiome analysis (days 0, 6, and 30) were frozen at −80°C. These samples were sent for microbiome analysis on dry ice to the Gastrointestinal Laboratory at Texas A&M University.

### 2.5 Microbiome analysis

#### 2.5.1 DNA extraction

DNA was extracted from an aliquot of 100 mg of each fecal sample using a MoBio Power soil DNA isolation kit (MoBio Laboratories) according to manufacturer's instructions. The bead‐beating step was performed on a homogenizer (FastPrep‐24; MP Biomedicals, Santa Ana, California) for 60 seconds at a speed of 4 m/s. Fecal DNA was stored at −80°C until further evaluation.

### 2.6 Quantitative PCR (qPCR)

For chosen bacterial taxa (ie, *Faecalibacterium* spp., *Turicibacter* spp., *Streptococcus* spp., *E. coli*, *Blautia* spp., *Fusobacterium* spp., and *Clostridium hiranonis*) and total bacteria, which are known to be altered in dogs with gastrointestinal disease, individual qPCR assays were performed and results used to calculate the recently described dysbiosis index (DI).^37^ The method, including the oligonucleotide sequence of the primers and the annealing temperatures were described elsewhere previously.^37^ A DI <0 reflects normobiosis, whereas a DI ≥2 dysbiosis, and values between 0 and 2 are considered to be equivocal. The abundances of *Clostridium perfringens* 16S rRNA gene, *C. perfringens* enterotoxin gene, *C. perfringens NetF* gene, *C. difficile* 16S rRNA gene, and *C. jejuni* gene in feces were analyzed by qPCR assays using the published oligonucleotide primers and assays.^38^, ^39^, ^40^, ^41^ PCR conditions were 95°C for 20 seconds, 40 cycles at 95°C for 5 seconds, and 10 seconds at the optimized annealing temperature. For probe‐based assays, the mastermix contained 10 μL of TaqMan reaction mixtures consisting of 5 μL of TaqMan Fast Universal PCR master mix (2×), No AmpErase UNG (Applied Biosystems), 0.4 μL of each primer (concentration: 400 nM), 0.2 μL of the probe (concentration: 200 nM), 1 μL of 1% bovine serum albumin (BSA, concentration: 0.1%), 1 μL of water, and 2 μL of DNA (1:10 or 1:100 dilution). For SYBR‐based assays, PCR procedures were performed at 95°C for 2 minutes, 40 cycles at 95°C for 5 seconds, and 10 seconds at the optimized annealing temperature with 10 μL of SYBR‐based reaction mixtures consisted of 5 μL of SsoFast EvaGreen supermix (Biorad Laboratories), 0.4 μL of each primer (concentration: 400 nM), 1 μL of 1% BSA (concentration: 0.1%), 1.6 μL of water, and 2 μL of DNA (1:10 or 1:100 dilution). The oligonucleotide sequences of the primers and probes, and the annealing temperatures can be found in Table S1.

### 2.7 Evaluation of the proportion of resistant fecal *E. coli*

One gram of feces was placed into a test tube and mixed with 9 mL of phosphate buffered saline (PBS; pH = 7.0). The mixture was homogenized for 5 minutes. The material was used in a 1:10 dilution series with PBS. Each 100‐μl aliquot of the dilutions was plated onto MacConkey‐Agar (MAC) plates containing no antibiotic and onto MAC plates mixed with ampicillin at a concentration of 32 μg/mL (MAC + AMP). This concentration was used based on the guidelines of the Clinical & Laboratory Standards Institute. Isolates that grew on MAC + AMP were considered to be cross‐resistant to ampicillin and amoxicillin. Plates were incubated for 20 to 24 hours at 37°C, and *E. coli* isolates from both plates were identified by Matrix Assisted Laser Desorption Ionization Time‐of‐Flight Mass Spectrometry (Bruker Microflex LT). The percentage of ampicillin‐resistant *E. coli* was calculated as follows: (number of isolates on MAC + AMP/number of isolates on MAC) × 100. The detection limit was set to 100 cfu/g. Resistant bacteria growing on the MAC + AMP were purified, and resistance of the isolates was confirmed by ampicillin (which is cross‐resistant to amoxicillin) disk diffusion susceptibility testing.

### 2.8 Statistical analyses

Power analysis determined that in order to detect a clinically relevant difference of 2 points in the CADS‐Index at day 3 between the AG and the PG, at least 8 dogs per group had to be included (with an estimated SD of 1.5, power of 80% and *P* < .05).

Statistical analyses were conducted with GraphPad Prism (GraphPad Prism c7.0, GraphPad Software, San Diego, California). Data were evaluated for normality by the D'Agonisto‐Pearson omnibus normality test. Differences in laboratory variables between groups were analyzed by unpaired *t* test with Welch's correction or the Mann‐Whitney *U* test. To avoid inflated type I error, Bonferroni correction was used. Differences in sex and last diarrheic event were evaluated with the Mann‐Whitney *U* test and the differences in the variables age, bodyweight, duration of diarrhea, diet change, and stressful event using an unpaired *t* test. The course of the CADS‐Index and those of individual variables, the DI, the included taxa (*Faecalibacterium* spp., *Turicibacter* spp., *Streptococcus* spp., *E. coli*, *Blautia* spp., *Fusobacterium* spp., and *C. hiranonis)*, total bacteria, *C. perfringens*, *C. perfringens* enterotoxin gene, *C. perfringens* NetF toxin, and *C. difficile* were analyzed by Kruskal‐Wallis test or ordinary ANOVA‐analysis (depending on normality) with Dunn's test for multiple comparison as a posttest for comparison between the 2 groups. The difference in the time to normalization of fecal consistency was evaluated with the Mann‐Whitney *U* test. Percentage of resistant *E. coli* were compared between the groups by a Mann‐Whitney *U* test and within group between different time points with the Wilcoxon matched‐pairs signed rank test. The adjusted significance level after Bonferroni correction for the laboratory variables was set at $\frac{0.05}{36}\;$ (= .0014). For all other statistical tests, the significance level was set at *P* = .05.

## 3 RESULTS

### 3.1 Study population

A total of 16 dogs fulfilled the inclusion criteria. Dogs were randomly assigned into the AG group (n = 8) and the PG group (n = 8). Age, sex, bodyweight, and prevalence of breeds were not different between the groups (Table 2). Median duration of diarrhea until presentation was 32 (range 4‐72) hours. Eleven of 16 dogs (69%) showed additional vomiting at least 1 time. Baseline laboratory variables of all dogs are presented in Table S2 showing no statistically significant differences between the groups after Bonferroni correction. Five of 16 owners (31%) described a stressful event for the dog in the last few days before presentation. One of 16 dogs (6%) was fed with a raw‐meat diet. Six of 16 dogs (37%) received table scraps as a treat and a dietary change had been made in 5 of 16 dogs (31%) before the diarrheic event. Other acute diseases in the last 30 days were documented in 5 of 16 dogs (31%) and chronic diseases in 6 of 16 dogs (37%). One of 16 dogs received levothyroxine as long‐term medication. Eleven of 16 dogs (69%) had a history of acute diarrheic events previously; however, the median interval to the last event was 2 (range 0.5‐10.0) months. No significant differences in variables from the dogs’ histories between groups were observed.

**TABLE 2:** Baseline variables in dogs with diarrhea

	AG (n = 8)		PG (n = 8)		*P*‐value
Sex	4 male, 4 female		3 male, 5 female		.88
Neutered/entire	5/3		2/6		.62
Breeds	Mixed breed (1), Australian Shepherd (1), Entlebucher Mountain Dog (1), German Hunting Terrier (1), Jack Russell Terrier (1), Maltese Dog (1), Miniature Poodle (1), Pug (1)		Mixed breed (3), Elo (1), Barbet (1), Labrador Retriever (1), Miniature Pinscher (1), Miniature Poodle (1)		

	Median	Range	Median	Range	
Bodyweight (kg)	9.1	5.6‐25.0	14.9	7.2‐29.5	.21
Age (years)	7.5	1.0‐11.0	5	1.0‐11.0	.30

Abbreviations: AG, amoxicillin‐clavulanic acid group; PG, placebo group.

### 3.2 Treatment efficacy

**FIGURE 1:**
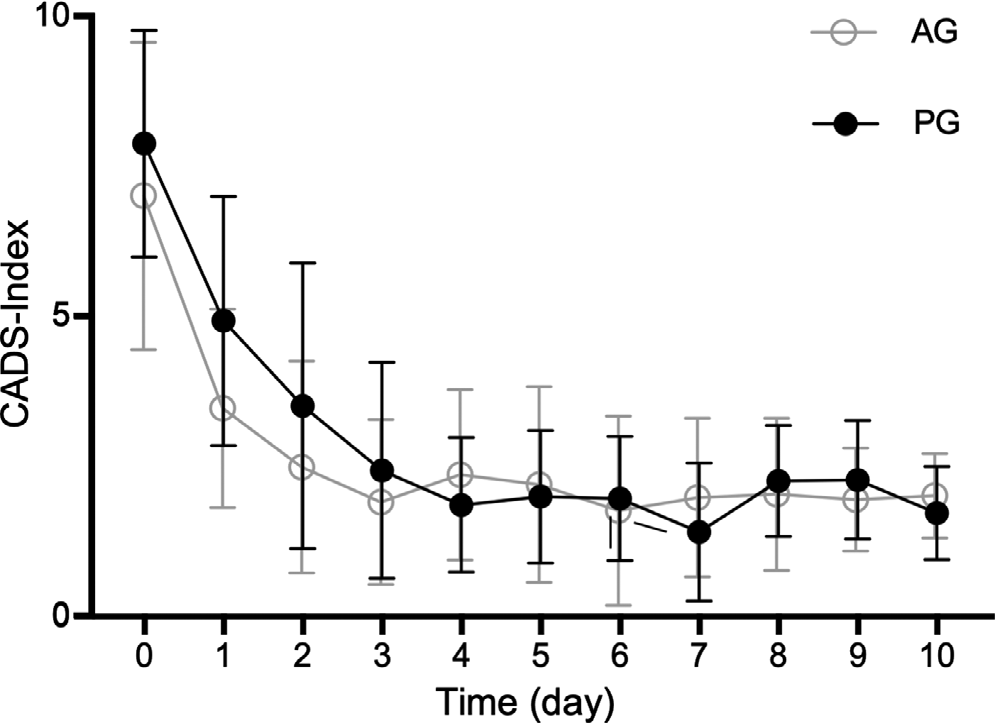
Clinical evaluation of clinical signs according to the canine acute diarrhea severity index (CADS index). The index includes the variables activity, appetite, vomiting (times/day), fecal consistency, and frequency of defecation (times/day). Each variable is scored from 0 to 3, and the sum of scores yields a total cumulative score. Dots show the mean, error bars show SD. No difference in the CADS‐Index on any day of the study period could be observed between AG and PG (*P* > .99). AG, amoxicillin‐clavulanic acid group; PG, placebo group

No statistically significant difference in the CADS‐Index (*P* > .99), and more specifically in fecal consistency (*P* > .99) on any day of the study period could be observed between the AG and the PG (Figure 1). On day 0, the day of inclusion, dogs were presented with a median CADS‐Index of 7 (range 4‐11; CI [4.9; 9.1]) in the AG group and 6 (range 5‐11; CI [6.3; 9.5]) in the PG group. There was no significant difference in the CADS‐Index between groups on day 0 (*P* > .99). On the last day of the study period (day 10) there was no significant difference of the CADS‐Index between the AG group (median: 2 (range: 1‐3; CI [1.4; 2.6]) and the PG group (median: 1.6 (range: 1‐3; CI [1.1; 2.4])) (*P* > .99): 2 dogs of the AG group and 1 dog of the PG group still had pasty feces upon conclusion of the study. All dogs in both groups reached normal clinical scores (CADS‐Index ≤3) 1 to 6 days (median 2 days) after presentation.

Regarding presence and frequency of vomiting, fecal frequency, activity, and appetite there was no significant difference between groups on all days of the study period.

The median time to normalization of fecal consistency was not statistically different between the AG (median: 1 day, range: 1‐6 days; CI [0.3; 2.3]) and PG (median: 2 days, range 1‐3 days; CI [1.2; 2.6]).

### 3.3 Microbiome analysis

**FIGURE 2:**
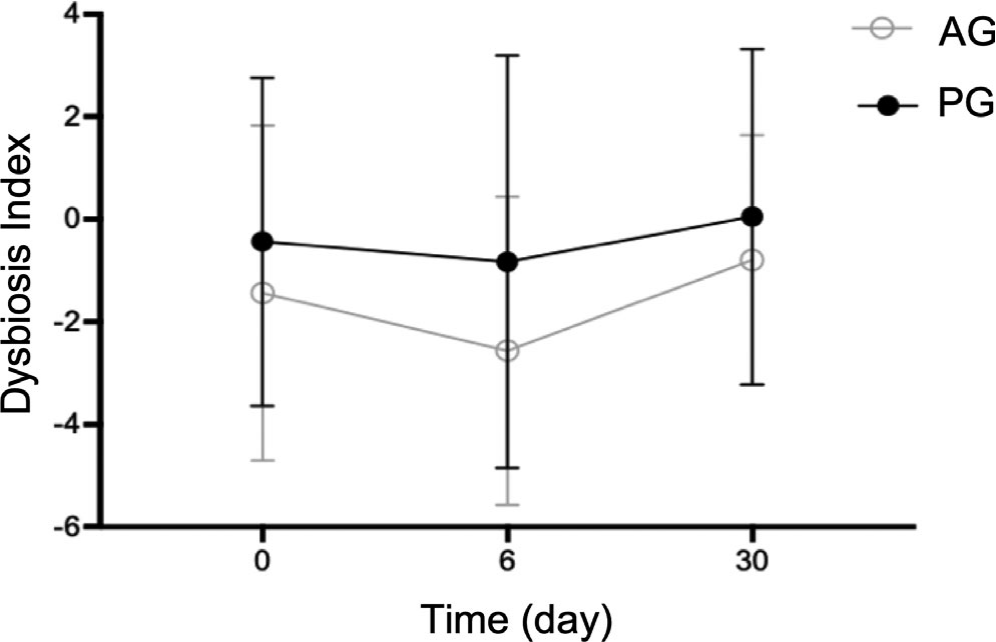
The dysbiosis index (DI) in dogs with diarrhea over time. A DI < 0 indicates normobiosis, whereas a DI ≥ 2 indicates dysbiosis. Dots show the mean, error bars show SD. There was no difference of the DI between the groups at any time point (*P* > .99). AG = amoxicillin‐clavulanic acid group. PG = placebo group

**FIGURE 3:**
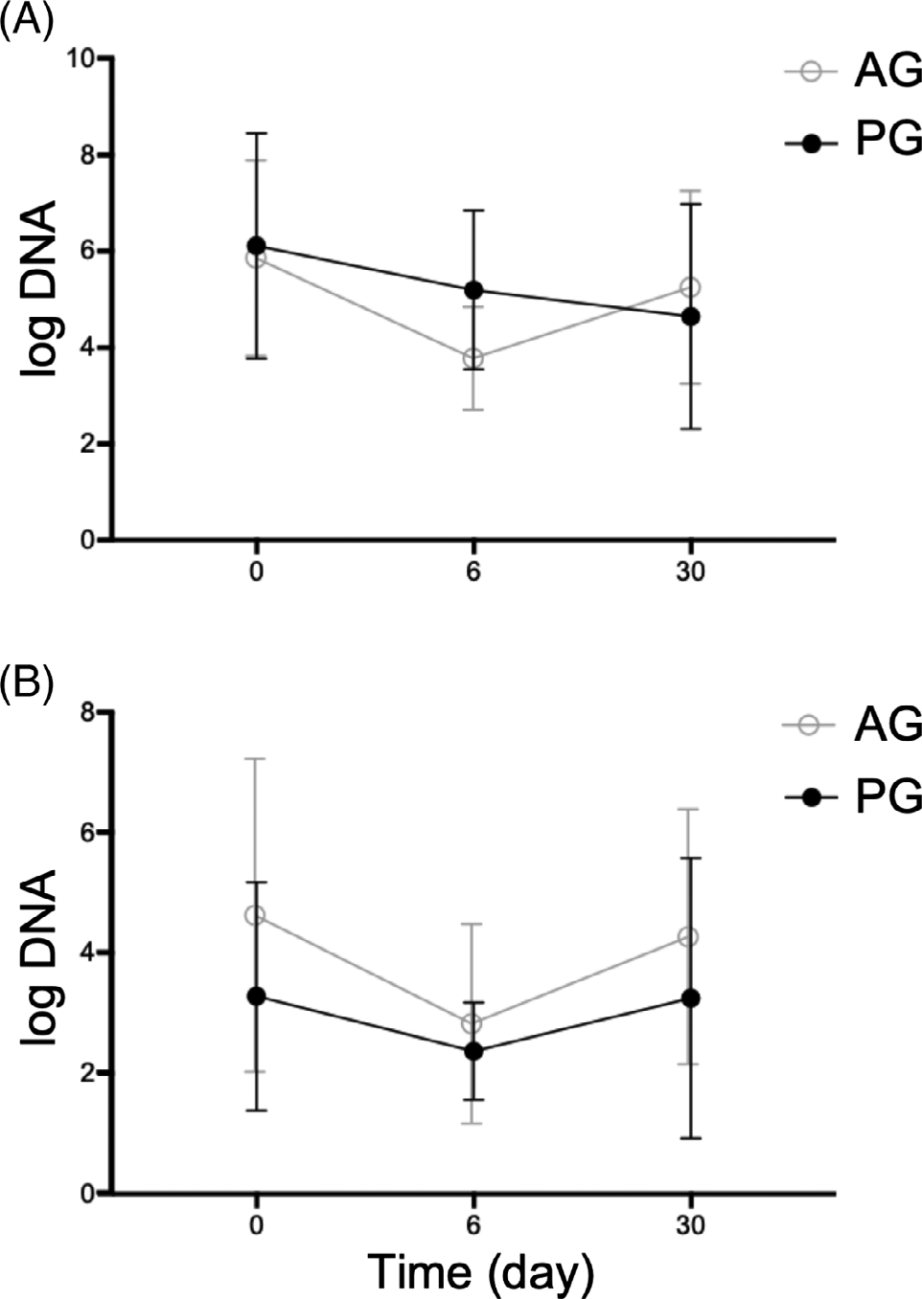
Abundance of *C. perfringens* (A) and *C. perfringens enterotoxin gene* (B). Dots show the mean, error bars show the SD. No difference in the fecal profiles for *C. perfringens* could be observed between the groups at any time point. AG, amoxicillin‐clavulanic acid group; PG, placebo group

There was no statistically significant difference of the DI (day 0 AG mean − 1,4 (SD 3.3; CI [−4.2; 1.3]); PG mean − 0.4 (SD 3.2; CI [−3.1; 2.2]); *P* > .99; day 6 AG mean − 2.6 (SD 3.0; CI [−5.1; 0.0]); PG mean − 0.8 (SD 4.0; CI [−4.2; 2.5]; *P* > .99; day 30 AG mean − 0.8 (SD 2.4; CI [−2.8; 1.2]); PG mean 0.1 (SD 3.3; CI [−2.7; 2.8]); *P* > .99) and investigated taxa between the groups at any time point (Figure 2, Table 3). An increased DI (> 2) was found in 7 dogs (AG: 3/8; PG: 4/8) for at least at 1 time point. Bacterial species considered as potentially enteropathogenic were evaluated in both groups. However, there was no significant difference in the fecal profiles for *C. perfringens. C. perfringens* enterotoxin gene was decreased on day 6 compared to day 0 with a rebound on day 30 in the AG without reaching significance (Figure 3). The *C. perfringens NetF* gene was found in feces of 2 dogs (1 dog on day 0 and 1 dog on day 30). In 3/8 dogs in the AG group on day 6 and in 1/8 dogs of the PG group on day 0 and 6, *C. difficile* strains could be detected, but there was no significant difference between groups at either time point. *Campylobacter jejuni* was not found in any sample.

**TABLE 3:** Dysbiosis index and the investigated phyla in dogs with diarrhea

	AG			PG		
	Day 0	Day 6	Day 30	Day 0	Day 6	Day 30
Dysbiosis index	−1.4 (3.3)	−2.6 (3.0)	−0.8 (2.4)	−0.4 (3.2)	−0.8 (4.0)	0.1 (3.3)
Total bacteria	10.8 (0.3)	10.8 (0.3)	10.8 (0.2)	10.8 (0.2)	10.7 (0.2)	10.5 (0.3)
*Faecalibacterium* spp.	5.1 (1.0)	5.2 (1.3)	6.0 (1.4)	4.7 (1.3)	4.6 (1.5)	5.1 (1.2)
*Turicibacter* spp.	6.1 (1.3)	5.7 (1.2)	5.6 (1.0)	5.8 (1.1)	5.8 (1.4)	6.4 (1.1)
*Streptococcus* spp.	4.4 (1.8)	3.8 (1.3)	5.2 (2.0)	4.6 (1.9)	4.5 (1.5)	5.5 (1.9)
*Escherichia coli*	6.7 (1.8)	5.9 (1.8)	6.0 (2.1)	7.1 (1.6)	6.4 (2.2)	6.7 (1.0)
*Blautia* spp.	10.1 (0.8)	10.4 (0.5)	10.4 (0.5)	10.0 (0.9)	9.9 (1.1)	9.9 (1.0)
*Fusobacterium* spp.	9.5 (1.1)	9.7 (0.5)	8.7 (1.5)	8.9 (0.8)	8.8 (1.1)	7.7 (1.1)
*Clostridium hiranonis*	4.9 (3.0)	5.0 (2.8)	5.0 (3.1)	4.8 (3.0)	5.1 (2.4)	5.2 (2.5)

*Note:* Values represent mean (SD) log DNA/g feces.

Abbreviations: AG, amoxicillin‐clavulanic acid group; PG, placebo group.

### 3.4 Evaluation of percentage of resistant fecal *E. coli*

**FIGURE 4:**
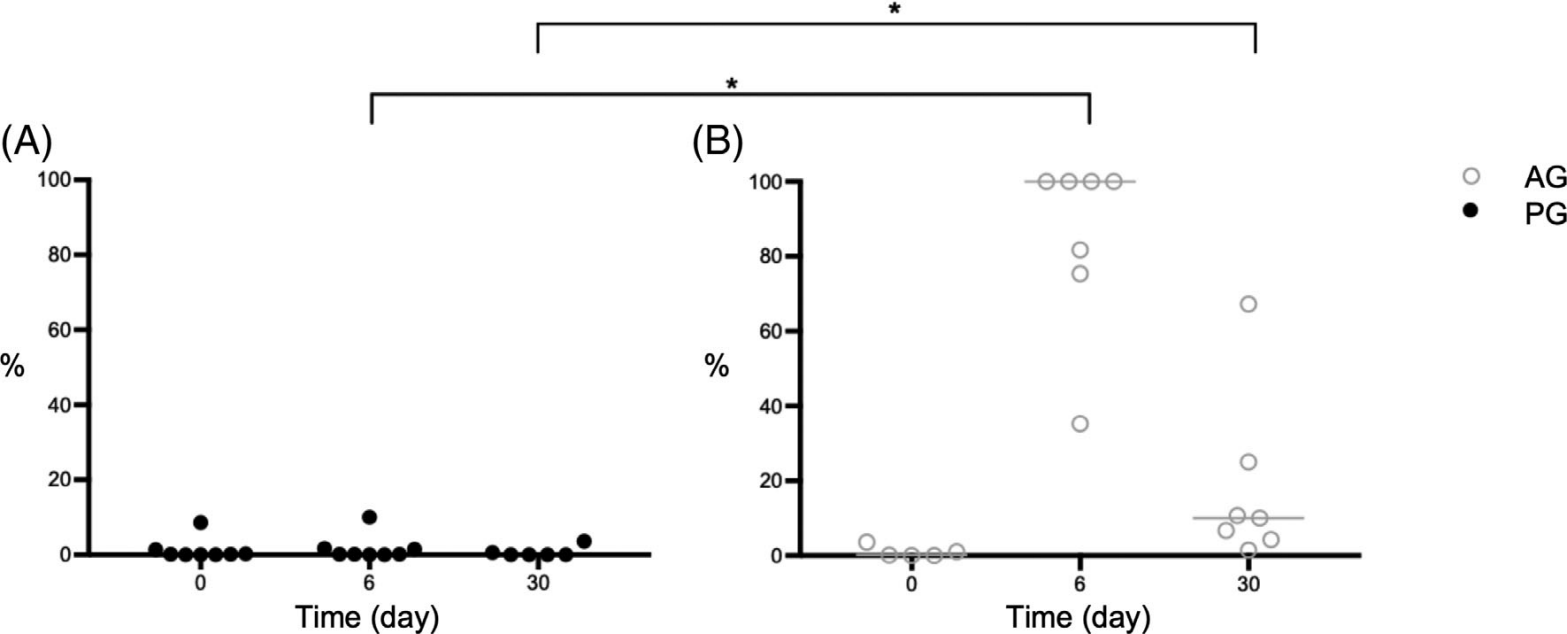
Percentages of amoxicillin‐resistant *E. coli* in diarrheic dogs treated with amoxicillin‐clavulanic acid (A) or placebo (B). Circles and filled in circles represent individual values for dogs of AG and PG, respectively. Bars show the median. *P < .05. A higher percentage of resistant *E. coli* could be observed in the AG group compared to the PG on day 6 (*P* < .001) during and on day 30 (*P* = .002) 3 weeks after antibiotic treatment. AG, amoxicillin‐clavulanic acid group; PG, placebo group

*E. coli* colonies were found in 44 of total 48 samples (92%). Forty of these 44 samples had at least 100 cfu/g resistant colonies. In 4 samples, resistant *E. coli* isolates were not detected. One of this 4 samples were cultured from a dog of the AG and 3 from dogs of the PG on day 30. On day 0, before the antibiotic treatment was started, the median percentage of ampicillin‐resistant *E. coli* was 0.2% (range 0%‐4%) in the AG group and 0.1% (range 0%‐9%) in the PG group with no significant difference between groups (*P* = .94). On day 6, the percentage of ampicillin‐resistant colonies was significantly higher in the AG than in the PG group. The median percentage of ampicillin‐resistant colonies rose to 100% in the AG group (range 35%‐100%), in the PG group the median was 0.2% (range 0%‐10%, *P* < .001). On day 30, 3 weeks after discontinuing antibiotic treatment, there was still a significant higher percentage of resistant *E. coli* in the AG group (median 10%; range 2%‐67%) than in the PG group (median 0%; range 0%‐4%; *P* = .002; Figure 4).

## 4 DISCUSSION

In this prospective, double‐blinded, placebo‐controlled study, clinical improvement of dogs with acute uncomplicated diarrhea was compared between dogs only treated with symptomatic treatment and those also treated with amoxicillin‐clavulanic acid. There was no statistically significant difference in the clinical course between groups at any time point. Oral treatment with amoxicillin‐clavulanic acid led to a significant increase of resistant *E. coli* isolates, but based on the DI, did not result in a significantly more prolonged dysbiosis compared with the PG.

The absence of a beneficial clinical effect of amoxicillin‐clavulanic acid treatment in dogs with uncomplicated AD is an important finding for clinicians, who routinely manage these cases on a daily basis. Amoxicillin‐clavulanic acid was chosen because beta‐lactam antibiotics are the most frequently used antimicrobials in gastrointestinal disease in dogs and cats.^1^, ^16^

It is estimated that currently more than half of dogs with AD are treated with antimicrobials, although a clinical benefit has not been proven so far.^1^, ^16^ This treatment approach is primarily based on the subjective impression of a more rapid clinical improvement with antibiotics. Furthermore, for several reasons there is a low threshold for using antibiotics in veterinary practice including diagnostic uncertainty, fear of clinical deterioration, time pressure, client expectations, and the general tradition to use antibiotics in dogs with diarrhea.^42^ This study should serve as a basis documenting that routine antibiotic treatment is not advantageous for dogs with uncomplicated AD. It underlines the recommendation in the ACVIM Consensus Statement of 2011 to administer antimicrobials only to dogs and cats with diarrhea that manifest systemic signs of illness (ACVIM consensus enteropathogenic bacteria).^10^, ^43^

No obvious negative short‐term clinical adverse effects of amoxicillin‐clavulanic acid treatment could be observed in this study, although it has been shown that administration of various antibiotics to healthy dogs and cats can cause diarrhea, which rapidly resolves after discontinuation of the antimicrobials. A current meta‐analysis in humans showed that about 10% of individuals with acute infectious enteritis develop chronic intestinal problems later in life, and treatment with antibiotics during the acute phase increases this risk.^44^ Further investigations are needed to evaluate the long‐term effects of antibiotics in dogs with AD on general health and more specifically on gut health.

The present study revealed that the most significant clinical improvement was observed within the first 2 days after the onset of clinical signs. This corresponds with findings of previous studies in which the duration of diarrhea was reported to be as 1 to 3 days,^8^ or less than 2 days.^9^ Dogs stayed clinically stable until the endpoint of the study (day 10). Three of 16 dogs still had pasty feces on day 10. This is in contradiction with a study describing a complete resolution of diarrhea in all dogs within a few days.^4^, ^9^ The different study design (retrospective versus prospective) might represent 1 explanation for a different assessment of the dog's status by the owner. In this context, the cause for the clinical changes are of interest. Around one third of the dogs had a stressful event, received table scraps, or underwent a diet change before presentation. These findings correspond with findings of previous studies, in which diet change and receiving treats were described as risk factors for acute gastrointestinal symptoms.^2^, ^9^

Based on the CADS‐Index, dogs in both groups were defined as moderately diseased. No clinically relevant changes on CBC or serum biochemistry profile could be observed in any dog. These facts can be explained by the exclusion of dogs with severe disease activity. This dog population was selected, because it was specifically aimed to assess if the typical case with uncomplicated diarrhea, which is seen by practitioners on a daily basis, benefits from antibiotic treatment.

A DI, based on the 7 bacterial taxa *Faecalibacterium*, *Turicibacter*, *Streptococcus*, *E. coli*, *Blautia*, *Fusobacterium*, and *C. hiranonis* and total bacteria, was determined in all dogs based on fecal samples collected on days 0, 6, and 30. This index was developed to quantify intestinal dysbiosis in dogs. Several studies revealed intestinal dysbiosis in dogs with AD and chronic enteropathies. In the present study, the median dysbiosis index for each treatment group was not significantly increased on day 0 in comparison with the reference range of the DI in either group. This means that in the present study population of dogs with uncomplicated AD, a significant dysbiosis could not be documented based on the DI. This finding suggests that in mild forms of AD the intestinal microbiome is not profoundly altered, which is in contrast to the significant dysbiosis documented in dogs with chronic enteropathies.^45^

Moreover, in the present study the treatment with amoxicillin‐clavulanic acid did not result in significant alterations in the DI or its included taxa during as well as 3 weeks after the treatment. In contrast, another study described a significant alteration in the intestinal microbiome in healthy dogs receiving amoxicillin.^26^, ^27^, ^28^ This discrepancy could partially be explained because the cited study was performed in healthy dogs, no control group for comparison of intestinal microbiota changes was used, and a huge interpatient variability was seen.

Recent investigations showed a profound effect of metronidazole and tylosin on the microbiota. Tylosin significantly increased the DI during treatment, and the abundance of *Faecalibacterium* was significantly decreased after treatment.^46^ Also, treatment with metronidazole in dogs leads to significant alterations in the intestinal microbiome with decreased abundances of the taxa *Bacteroidaceae*, *Clostridiaceae*, *Fusobacteriaceae*, *Lachnospiraceae*, *Ruminococcaceae*, *Turicibacteraceae*, and *Veillonellaceae* and increased abundances of *Bifidobacteriaceae*, *Enterobacteriaceae*, *Enterococcaceae*, and *Streptococcaceae*.^27^

Based on the discrepancy of the microbiome alterations between different antimicrobials, further investigations comparing the effect of several antimicrobials on a defined population of dogs in 1 study would be desirable. One possible explanation for the lack of detectable dysbiosis in the AG is that the diarrheal process itself could have reduced exposure of the gut microbiome to amoxicillin‐clavulanic acid; however, given the significant effect of antibiotic treatment on amoxicillin resistance in *E. coli*, this possibility can be eliminated. In contrast, in a study of healthy cats treated with amoxicillin‐clavulanic acid, the cats showed significant changes of the intestinal microbiome,^19^ consequently species‐dependent differences might be possible.

*C. perfringens* can be found as a normal component of the intestinal tract in healthy dogs. However, clostridial overgrowth plays an important role in dogs with acute hemorrhagic diarrhea syndrome (AHDS).^47^ Applying a quantitative PCR protocol, no significant differences in the abundance of *C. perfringens* in dogs of both treatment groups on day 0 were detected. A decrease in both *C. perfringens* and its enterotoxin‐encoding strains on day 6 and a rebound on day 30 was observed in the AG group; however, without reaching significance in comparison with the PG. Similar findings were shown in a cat population also treated with amoxicillin‐clavulanic acid.^19^ Thus, amoxicillin‐clavulanic acid appears to be effective in reducing the number of clostridial strains. However, this effect is only short‐lived, because the abundance of *C. perfringens* has been reported to return to baseline values a few weeks after discontinuing antimicrobial treatment.^48^ A correlation of clinical signs with the abundance of *C. perfringens* strains was not observed in the present study. The *C. perfringens NetF* toxin gene was found in 2 dogs (corresponding to 4% of the fecal samples) and therefore we conclude it is unlikely to have played a substantial role in causing diarrhea in the present study population. In previous studies, a higher abundance of *C. perfringens*, encoding for *NetF* toxin gene was detected in dogs AHDS,^49^, ^50^ but *C. perfringens* encoding for *NetF* toxin gene decreased within a few days even without the use of antibiotics.^51^ A recent paper showed a prevalence of 48.1% in dogs with acute hemorrhagic diarrhea whereas only 12.1% of the healthy dogs carried the *NetF* gene.^52^

*C. difficile* is known to induce pseudomembranous colitis and is associated with significant morbidity and mortality in humans.^53^ Several studies showed that antibiotic use and more specifically the application of penicillin, is a major risk factor for the development of *C. difficile* infections.^54^, ^55^ The role of *C. difficile* in the etiology of canine diarrhea is still unclear. However, some studies showed a slightly higher prevalence of *C. difficile* in dogs with diarrhea compared to healthy dogs.^47^, ^56^ In the present study, no dog of the AG group was positive for *C. difficile* by qPCR on day 0, whereas on day 6, 38% of the dogs tested positive. Although, the results did not reach significance, this finding suggests that destruction of the protective intestinal microbiota by amoxicillin‐clavulanic acid may promote the growth of potentially pathogenic bacteria in the intestinal tract. Further studies are needed to evaluate the role of antibiotics in the emergence of larger numbers of *C. difficile* in dogs and cats.

The third goal of the study was to evaluate the impact of amoxicillin‐clavulanic acid on the amount of amoxicillin‐resistant fecal *E. coli*. This bacterial strain was chosen as a marker species because 98% to 99% of dogs harbor *E. coli* in their intestines.^57^, ^58^ Although *E. coli* are normal commensal bacteria in the canine intestinal tract and most strains are nonpathogenic, *E. coli* is frequently found in urinary tract infections and has the ability to cause wound infections.^59^ Every dog in our study treated with amoxicillin‐clavulanic acid for 6 days developed significant populations of amoxicillin‐resistant *E. coli*. In most dogs even 100% of their *E. coli* had developed resistance. This is in line with recent investigations showing that amoxicillin‐resistant *E. coli* can be detected in 20.2% to 78.0% of individuals treated with this potentiated penicillin derivates.^60^, ^61^ Antibiotics can have a marked effect on the development of antimicrobial‐resistant isolates.^62^, ^63^ Most bacterial clones are killed or the growth stops under antimicrobial therapy; however, there are invariably nonsusceptible subpopulations that survive the antimicrobial challenge, because of intrinsic and acquired antimicrobial resistance or both.^64^ These resistant isolates then flourish, because of reduced competition from susceptible strains. Antimicrobial‐resistant bacterial strains can not only complicate infections in individuals harboring these bacteria, they can also be transmitted, directly or indirectly, to other animals and humans. Antimicrobial resistance genes—sometimes several at once—can also be transferred to other bacterial species via plasmids, transposable elements or phages.^65^ Resistant bacterial strains can complicate infections in individuals harboring these bacteria (eg, ascending *E. coli* cystitis during antibiotic treatment) and can spread into the environment and affect other otherwise healthy individuals. In this context, *E. coli* is only a marker species and it must be assumed that many other bacterial groups respond to the selection pressure of antimicrobial treatment in a similar way. Veterinarians must be aware of their effect on the global emergence of drug‐resistant infections by prescribing broad‐spectrum antibiotics in dogs with uncomplicated disease, especially when indication for antibiotic treatment is lacking.

Moreover, amoxicillin‐resistant *E. coli* could still be isolated from dogs treated with antibiotics 21 days after treatment. This emphasizes that the intestinal tract acts as a reservoir for resistant bacteria long after treatment has been stopped. Different studies suggest that high levels of resistance genes can still be found up to 4 years after antibiotic exposure.^66^, ^67^ This emphasizes once more the importance of prudent antimicrobial usage in order to prevent spread of antibiotic resistance.

There are limitations of the study. First of all, owners gave the treatments at home. Although owners were trained in administering the capsules according to the schedule and to report if capsule administration was not possible, it cannot be completely ruled out that on some occasions, treatments were not administered. Moreover, clinical disease activity of dogs was scored by the owner themselves. Although some variables included in scores that can be objectively assessed (eg, defecation frequency, fecal consistency based on fecal scoring system), some variables (eg, activity) are relatively subjective. Clinical impression also depends on the time spent with the dog, which differs between owners. Moreover, all enrolled dogs are from the same geographical region, which makes generalization impossible. Furthermore, complete fecal microbiota sequencing was not performed. Therefore, for the purpose of the microbiome analysis it cannot be ruled out that a different method would have been able to detect changes in the composition of the intestinal microbiome between dogs treated with and without amoxicillin‐clavulanic acid. No specific mechanism of resistance was investigated in this study. The main limitation of this study is the small number of dogs in both treatment groups. Sample‐size calculation was based on clinician's opinion, because comparable studies were lacking at the time when the study‐protocol was designed. It is impossible to make clear recommendations based on the present study alone. Together with other published data, the present study should make a contribution toward setting up guidelines on the usage of antimicrobials in AD. However, the difference in development of bacterial resistance to amoxicillin was very clear and statistically significant and all dogs improved clinically within the study period. Thus, it seems unlikely that the results would have changed significantly with a larger group.

## CONFLICT OF INTEREST DECLARATION

Authors declare no conflict of interest.

## OFF‐LABEL ANTIMICROBIAL DECLARATION

Authors declare no off‐label use of antimicrobials.

## INSTITUTIONAL ANIMAL CARE AND USE COMMITTEE (IACUC) OR OTHER APPROVAL DECLARATION

Prospective collection and analysis of canine fecal samples was approved by the Ethics Committee of the Centre of Veterinary Medicine, Ludwig‐Maximilian‐University, Munich, Germany (reference 68‐19‐05‐2016).

## HUMAN ETHICS APPROVAL DECLARATION

Authors declare human ethics approval was not needed for this study.

## Supporting information


**Supplementary Table 1** Oligonucleotides primers/probes used in this study.

**Supplementary Table 2** Laboratory results in dogs with diarrhea
