# Supplementary material for: Effect of amoxicillin‐clavulanic acid on clinical scores, intestinal microbiome, and amoxicillin‐resistant Escherichia coli in dogs with uncomplicated acute diarrhea
Source: J Vet Intern Med. 2020 Apr 23;34(3):1166–76. doi: 10.1111/jvim.15775 (PMC7255678; doi:10.1111/jvim.15775)
Supplement: Supplementary file 1 — Supplementary Table 1 Oligonucleotides primers/probes used in this study. [file JVIM-34-1166-s001.pdf]

Supplementary Table 1. Oligonucleotides primers/probes used in this study.

qPCR primers/probe	Sequence (5’- 3’)	Target	Annealing (°C)	Reference
Forward	GAAGGCGGCCTACTGGGCAC	*Faecalibacterium*	60	[1]
Reverse	GTGCAGGCGAGTTGCAGCCT			
Forward	KGGGCTCAACMCMGTATTGCGT	Fusobacteria	51	[2]
Reverse	TCGCGTTAGCTTGGGCGCTG			
Forward	TCTGATGTGAAAGGCTGGGGCTTA	*Blautia*	56	[2]
Reverse	GGCTTAGCCACCCGACACCTA			
Forward	CCTACGGGAGGCAGCAGT	Universal Bacteria	59	[3]
Reverse	ATTACCGCGGCTGCTGG			
Forward	CAGACGGGGACAACGATTGGA	*Turicibacter*	63	[2]
Reverse	TACGCATCGTCGCCTTGGTA			
Forward	GTTAATACCTTTGCTCATTGA	*E. coli*	55	[4]
Reverse	ACCAGGGTATCTAATCCTGTT			
Forward	AGTAAGCTCCTGATACTGTCT	*C. hiranonis*	50	[5]
Reverse	AGGGAAAGAGGAGATTAGTCC			
Forward	TTATTTGAAAGGGGCAATTGCT	*Streptococcus*	54	[6]
Reverse	GTGAACTTTCCACTCTCACAC			
Forward	AACTATAGGAGAACAAAATACAATAG	*C. perfringens* enterotoxin gene	55	[7]
Reverse	TGCATAAACCTTATAATATACATATTC			
Probe	FAM-TCTGTATCTACAACTGCTGGTCCA-TAMURA			
Forward	CGCATAACGTTGAAAGATGG	*C. perfringens* 16S rRNA gene	58	[8]
Reverse	CCTTGGTAGGCCGTTACCC			
Probe	FAM-TCATCATTCAACCAAAGGAGCAATCC-TAMURA			
Forward	AACAATATGTACAGGTATAACT	*C. perfringens netF* toxin gene	55	[9]
Reverse	TTGATAGGTATAATATGGTTCT			
Forward	TTGAGCGATTTACTTCGGTAAAGA	*C. difficile*	61	[10]
Reverse	TGTACTGGCTCACCTTTGATATTCA			
Forward	5’-GAGCTTTCAAGCCCTTATATCC-3’	*C. jejuni*	62.5	[11]
Reverse	5’-AAGAACACCGCGAAGTTTATTT-3’			

REFERENCES

1. Garcia-Mazcorro JF, Suchodolski JS, Jones KR, et al. Effect of the proton pump inhibitor omeprazole on the gastrointestinal bacterial microbiota of healthy dogs. *FEMS Microbiol Ecol.*2012;80(3):624-636.
2. Suchodolski JS, Markel ME, Garcia-Mazcorro JF, et al. The fecal microbiome in dogs with acute diarrhea and idiopathic inflammatory bowel disease. *PloS one*. 2012;7(12):e51907.
3. Lubbs D, Vester B, Fastinger N, Swanson KS. Dietary protein concentration affects intestinal microbiota of adult cats: a study using DGGE and qPCR to evaluate differences in microbial populations in the feline gastrointestinal tract. *J Anim Physiol Anim Nutr (Berl).* 2009;93(1):113-121.
4. Malinen E, Rinttilä T, Kajander K, et al. Analysis of the fecal microbiota of irritable bowel syndrome patients and healthy controls with real-time PCR. *Am J Gastroenterol.* 2005;100(2):373.
5. Kitahara M, Sakamoto M, Benno Y. PCR detection method of Clostridium scindens and C. hiranonis in human fecal samples. *Microbiol Immunol.* 2001;45(3):263-266.
6. Furet JP, Quénée P, Tailliez P. Molecular quantification of lactic acid bacteria in fermented milk products using real-time quantitative PCR. *Int J Food Microbiol.* 2004;97(2):197-207.
7. Gurjar A, Hegde N, Love B, Jayarao B. Real-time multiplex PCR assay for rapid detection and toxintyping of Clostridium perfringens toxin producing strains in feces of dairy cattle. *Mol Cell Probes*. 2008;22(2):90-95.
8. Wise MG,Siragusa GR. Quantitative detection of Clostridium perfringens in the broiler fowl gastrointestinal tract by real-time PCR. Appl. *Environ. Microbiol*. 2005;71(7):3911-3916.
9. Gohari IM, Parreira VR, Nowell VJ, et al. A novel pore-forming toxin in type A Clostridium perfringens is associated with both fatal canine hemorrhagic gastroenteritis and fatal foal necrotizing enterocolitis. *PloS one.* 2015;10(4):e0122684.
10. Penders J, Vink C, Driessen C, et al. Quantification of Bifidobacterium spp., Escherichia coli and Clostridium difficile in faecal samples of breast-fed and formula-fed infants by real-time PCR. F*EMS Microbiol Lett.* 2005;243(1):141-147.
11. Chaban B, Musil KM, Himsworth CG, Hill JE. Development of cpn60-based real-time quantitative PCR assays for the detection of 14 Campylobacter species and application to screening of canine fecal samples. *Appl. Environ. Microbiol.* 2009;75(10):3055-3061.
