# Supplementary material for: Effect of amoxicillin‐clavulanic acid on clinical scores, intestinal microbiome, and amoxicillin‐resistant Escherichia coli in dogs with uncomplicated acute diarrhea
Source: J Vet Intern Med. 2020 Apr 23;34(3):1166–76. doi: 10.1111/jvim.15775 (PMC7255678; doi:10.1111/jvim.15775)
Supplement: Supplementary file 2 — Supplementary Table 2 Laboratory results in dogs with diarrhea [file JVIM-34-1166-s002.pdf]

Supplementary Table 2. Laboratory results in dogs with diarrhea

		AG (n=8)		PG (n=8)		p-value
		Median	Range	Median	Range	
urea	mg/dl	21	12-58	20	8-37	.362
creatinine	mg/dl	0.6	0.5-1.8	0.95	0.5-1.2	.235
SDMA	µg/dl	10.5	5-17	12.5	9-19	.191
sodium	mmol/l	146.5	145-151	143.5	141-147	.002
chloride	mmol/l	113.5	106-118	110.5	107-115	.192
potassium	mmol/l	4.3	3.9-4.9	4.6	3.8-4.8	.904
phosphate	mmol/l	1.2	0.0-1.8	1.35	0.9-2.0	.482
total bilirubin	mg/dl	0.2	0.2-0.3	0.2	0.2-0.3	>.999
ALT	U/l	54.5	18-132	45	28-79	.386
ALP	U/l	36	17-334	43.5	17-211	.643
gamma-GT	U/l	4.5	3-11	4	3-6	.178
AST	U/l	27.5	20-35	35.5	16-45	.088
GLDH	U/l	3.5	2-8	3.5	2-7	.703
total protein	g/dl	6.1	5.4-6.7	6.3	5.4-7.5	.740
albumin	g/dl	3.1	2.3-3.6	3.15	2.5-3.4	.890
globulin	g/dl	3.1	2.5-3.6	3.25	2.4-4.1	.516
glucose	mg/dl	92.5	51-105	95.5	55-116	.458
alpha-amylase	U/l	723	407-1379	729	510-1106	.813
lipase	U/l	104	36-224	37	16-137	.048
cholesterol	mg/dl	252.5	148-317	260.5	188-502	.645
fructosamine	µmol/l	252	226-310	286.5	207-378	.505
creatine kinase	U/l	90	60-302	91.5	60-204	.590
calcium	mmol/l	2.4	2.4-2.6	2.425	2.3-2.8	.306
magnesium	mmol/l	0.8	0.8-1.0	0.8	0.7-1.0	.770
triglyceride	mg/dl	61	27-228	61.5	26-163	.778

RBC	x 10^{12}/L	6.7	4.7-8.6	7.45	6.6-8.4	.066
HGB	mmol/L	16.15	11.2-20.5	16,10	10.6-19.1	.165
PCV	%	47.4	36.7-60.0	49.4	46.0-54.2	.302
WBC	x 10^{9}/L	9.7	5.3-11.6	9.75	5.8-27.1	.489
basophils	/µl	0	0-1.0	0	0	>.999
eosinophils	/µl	319.5	97-1043	256.5	0-890	.879
neutrophils	/µl	6475	3900-9080	6340	4228-21122	.645
bands	/µl	0	0-1275	0	0	>.999
lymphocytes	/µl	1259	649-2434	1751	730-3002	.254
monocytes	/µl	600	194-1159	567	77-3250	.505
platelets	x 10^{9}/L	258	121-373	176.5	103-307	.281

AG = amoxicillin clavulanic acid group; PG = placebo group; SDMA = symmetric dimethylarginine assay; ALT= alanine transaminase; ALP= alkaline phosphatase; gamma-GT= gamma-glutamyl transferase; AST= aspartate aminotransferase; GLDH= glutamate dehydrogenase; RBC= red blood cells; HGB= hemoglobin; WBC= white blood cells; * significance level was set at .0014 after Bonferroni correction
